# Pre-Failure Strain Localization in Siliclastic Rocks: A Comparative Study of Laboratory and Numerical Approaches

**DOI:** 10.1007/s00603-024-04025-y

**Published:** 2024-06-22

**Authors:** Patrick Bianchi, Paul Antony Selvadurai, Luca Dal Zilio, Antonio Salazar Vásquez, Claudio Madonna, Taras Gerya, Stefan Wiemer

**Affiliations:** 1 Swiss Seismological Service, ETH Zurich, Zurich, Switzerland; 2 Institute of Geophysics, ETH Zurich, Zurich, Switzerland; 3 Earth Observatory of Singapore, Nanyang Technological University, Singapore, Singapore; 4 Asian School of the Environment, Nanyang Technological University, Singapore, Singapore; 5 University of Applied Sciences of Eastern Switzerland, Rapperswil, Switzerland; 6 Geological Institute, ETH Zurich, Zurich, Switzerland

**Keywords:** Strain localization, Preparatory processes, Acoustic emissions, Distributed strain sensing with optical fibers, Continuum-based numerical modeling

## Abstract

We combined novel laboratory techniques and numerical modeling to investigate (a)seismic preparatory processes associated with deformation localization during a triaxial failure test on a dry sample of Berea sandstone. Laboratory observations were quantified by measuring strain localization on the sample surface with a distributed strain sensing (DSS) array, utilizing optical fibers, in conjunction with both passive and active acoustic emission (AE) techniques. A physics-based computational model was subsequently employed to understand the underlying physics of these observations and to establish a spatio-temporal correlation between the laboratory and modeling results. These simulations revealed three distinct stages of preparatory processes: (i) highly dissipative fronts propagated towards the middle of the sample correlating with the observed acoustic emission locations; (ii) dissipative regions were individuated in the middle of the sample and could be linked to a discernible decrease of the P-wave velocities; (iii) a system of conjugate bands formed, coalesced into a single band that grew from the center towards the sample surface and was interpreted to be representative for the preparation of a weak plane. Dilatative lobes at the process zones of the weak plane extended outwards and grew to the surface, causing strain localization and an acceleration of the simulated deformation prior to failure. This was also observed during the experiment with the strain rate measurements and spatio-temporally correlated with an increase of the seismicity rate in a similar rock volume. The combined approach of such laboratory and numerical techniques provides an enriched view of (a)seismic preparatory processes preceding the mainshock.

## Highlights

- The combination of novel laboratory and numerical techniques allowed us to detect preparatory processes prior to failure.
- The employment of distributed strain sensing with optical fibers was successful in imaging strain localization preceding failure.
- The simulated dissipation of mechanical energy correlated with the observations of strain localization occurring during the experiment.

## Introduction

Forecasting destructive earthquakes continues to be a significant challenge due to the complex physical processes governing the nucleation of seismic events. Both field (Gulia and Wiemer 2019; Campillo and Paul 2003) and laboratory (Scholz 1968; Meredith et al. 1990; Yamashita et al. 2021) studies have demonstrated that spatio-temporal variations of seismic or aseismic quantities (e.g., statistical analyses of earthquake catalogues or wave velocity perturbations) occur in proximity of the mainshock hypocenter. These variations are attributed to the preparation phase of large earthquakes that may involve the localization of strain or preslip (Bürgmann 2014; Kato and Ben-Zion 2021). Two end-member hypotheses exist to describe strain localization preceding earthquakes. The first has notably been developed in theories related to frictional stability, it describes preparatory processes along infinitesimally thin, pre-existing discontinuities in the form of slip and has been successful in investigating physical processes related to earthquake nucleation due to frictional instabilities (Dieterich 1979, 1992; Ruina 1983; Rice 1993; Lapusta and Rice 2003; Rubin and Ampuero 2005). The second approach focuses on how preparatory processes in the form of strain localization lead to the formation of rupture planes (Griggs and Handin 1960; Mogi 1967), which are inherently complex and non-linear (e.g., Mitchell and Faulkner 2009; Faulkner et al. 2010; Behr and Bürgmann 2021; Cocco et al. 2023). In this study we focused on the second approach. Understanding the physical mechanisms responsible for spatio-temporal strain localization and accelerated deformation preceding large seismic events and developing tools capable of simulating these processes would enhance our ability to better forecast earthquakes in both space and time.

In the field, direct observations of preparatory processes related to the localization of strain and rock damage are challenging. However, the spatio-temporal evolution of small earthquakes provides invaluable insights into these processes, offering a critical perspective that is applicable across a range of spatial scales, from underground laboratory settings (e.g., Guglielmi et al. 2015; De Barros et al. 2018; Villiger et al. 2020) to real faults (e.g., Ross et al. 2019). A key aspect of this analysis is the b-value of the well-known Gutenberg–Richter law (Gutenberg and Richter 1944), which serves as an important by-product in understanding the dynamics and scale of seismic events. This parameter reflects the amount of small-to-large earthquakes and is the current objective of many research efforts, since it has been shown to decrease in proximity to a mainshock and its variation reflects preparatory processes to the larger earthquake (e.g., Gulia et al. 2016; Gulia and Wiemer 2019). Emphasizing the spatio-temporal aspect underscores the universal relevance of these studies in deciphering the complex mechanics of rock failure and seismic activity. Analytical studies have presented mathematical formulations to relate the seismicity detected in a rock volume to the degree of damage, which was identified by variations of the elastic moduli in the full moment-rate tensor (Ben-Zion and Ampuero 2009; Ben-Zion and Lyakhovsky 2019). Deformation measurements, based on geodetic data, have revealed that preparatory processes in the form of aseismic, slow deformation were taking place before the mainshock (e.g., Kato et al. 2012; Obara and Kato 2016). The zones interested by these precursory aseismic processes have been linked to increases in seismic activity (i.e., fast deformation) preceding the mainshock (Kato et al. 2016), as evidenced by, e.g., the preseismic phases of the Mw 9.0 Tohoku-Oki earthquake (Kato et al. 2012) and the Mw 8.2 Iquique earthquake (Kato and Nakagawa 2014; Kato et al. 2016). Both seismic and aseismic deformations are responsible for the dissipation of mechanical energy in the form of shear heating during preparatory processes (e.g., Rice 2006). Investigating the interplay between these two deformation forms is thus crucial to better capture the physical mechanisms controlling strain localization prior to the mainshock.

Strain localization is not only investigated at the field scale but also in rock samples during typical deformation laboratory experiments at the centimeter scale. Similar preparatory processes can be studied through the recording of laboratory seismicity often referred to as acoustic emissions (AEs) (e.g., Hardy 2003; Lei and Ma 2014). AEs offer insight into the development of microfractures, damage and the eventual coalescing of a macrofracture that results in dynamic failure (Sondergeld and Estey 1981; Nishizawa et al. 1984). Relationships such as the Gutenberg–Richter law (Mogi 1962; Scholz 1968), Omori’s law (Hirata et al. 1987; Marty et al. 2023) or the relation between fracture energy and slip (Selvadurai 2019) represent promising links to large scale natural processes but would benefit from more accurate understanding of the physical mechanisms producing these events. Baud et al. (2004) conducted conventional triaxial tests on high-porosity samples of different sandstone typologies confined at pressures ranging from 40 to 395 MPa. By combining observations retrieved from mechanical deformation, microstructural and AE data, they demonstrated that strain localization in the form of compaction bands and high-angle shear bands occurs in high-porous lithologies. Grain crushing and pore collapses induced by grain scale microcracking (e.g., Menéndez et al. 1996) were individuated to be the dominant processes leading to the sample compaction during triaxial studies based on a combination of AE data and micromechanical analyses performed on a wide range of sandstones (Zhang et al. 1990a, b, c).

Laboratory experiments have been instrumental in our understanding of earthquakes and fault mechanics (Scholz 2019). However, the physical mechanisms controlling these phenomena still remain largely elusive. To bridge this gap, advanced monitoring techniques have emerged, enhancing the detection and analysis of preparatory processes. Dynamic X-ray tomography (McBeck et al. 2021b, 2022a, b; Cartwright-Taylor et al. 2022) and distributed strain sensing (DSS) with optical fibers (Salazar Vásquez et al. 2022a, b; Bianchi et al. 2022), for example, have significantly increased the spatial resolution at which these processes are observed, revealing insights into slow and aseismic deformations that traditional methods might miss. These novel sensors complement conventional seismic monitoring and have become invaluable in capturing the complex interplay between seismic and aseismic behaviors. Furthermore, recent advancements in multi-physics, fully-coupled hydro-mechanical computational methods (e.g., Dal Zilio et al. 2018, 2019; Gerya 2019; Petrini 2019; Petrini et al. 2020; Dal Zilio et al. 2022; Dal Zilio and Gerya 2022), have opened new avenues for exploring these intricate phenomena. These sophisticated models allow for a deeper investigation into the nonlinear processes underlying the preparatory phases of earthquakes, providing a more comprehensive understanding of strain localization and its implications for seismic activity. Together, these laboratory innovations and computational tools represent a significant leap forward in our ability to decipher the subtle precursors of seismic events but only few recent, partial attempts have been conducted to address this challenge in the past years (e.g., Bianchi et al. 2022).

Driven by the necessity of better understanding how (a)seismic preparatory processes in the form of strain localization could lead to the increase of seismic activity and subsequent mainshock, here we present a study of the localization of deformation during a triaxial experiment on a dry sample of Berea sandstone. To shed light on these partially elusive processes, we combined state-of-the-art laboratory and numerical techniques. Piezoelectric transducers (PZTs) were employed to localize the AEs and to construct a homogeneous time-varying velocity model used to track velocity variations associated to damage within the sample. Furthermore, novel surface strain monitoring techniques (DSS with optical fibers) allowed us to quantitatively image the bulk deformation during the test and advanced physics-based numerical models (H-MEC, Dal Zilio et al. 2022) were used to investigate processes occurring within the sample. To our knowledge, no numerical tools of such kind have ever been tested against such extensive deformation datasets at this high level of details. Numerous correlations among the laboratory and numerical observations obtained were presented and thoroughly discussed, offering novel insights into the topic of pre-failure strain localization and accelerated deformation with potential significance for the larger reservoir or field scales.

## Materials and Methods

### Rock Sample

**Fig. 1:**
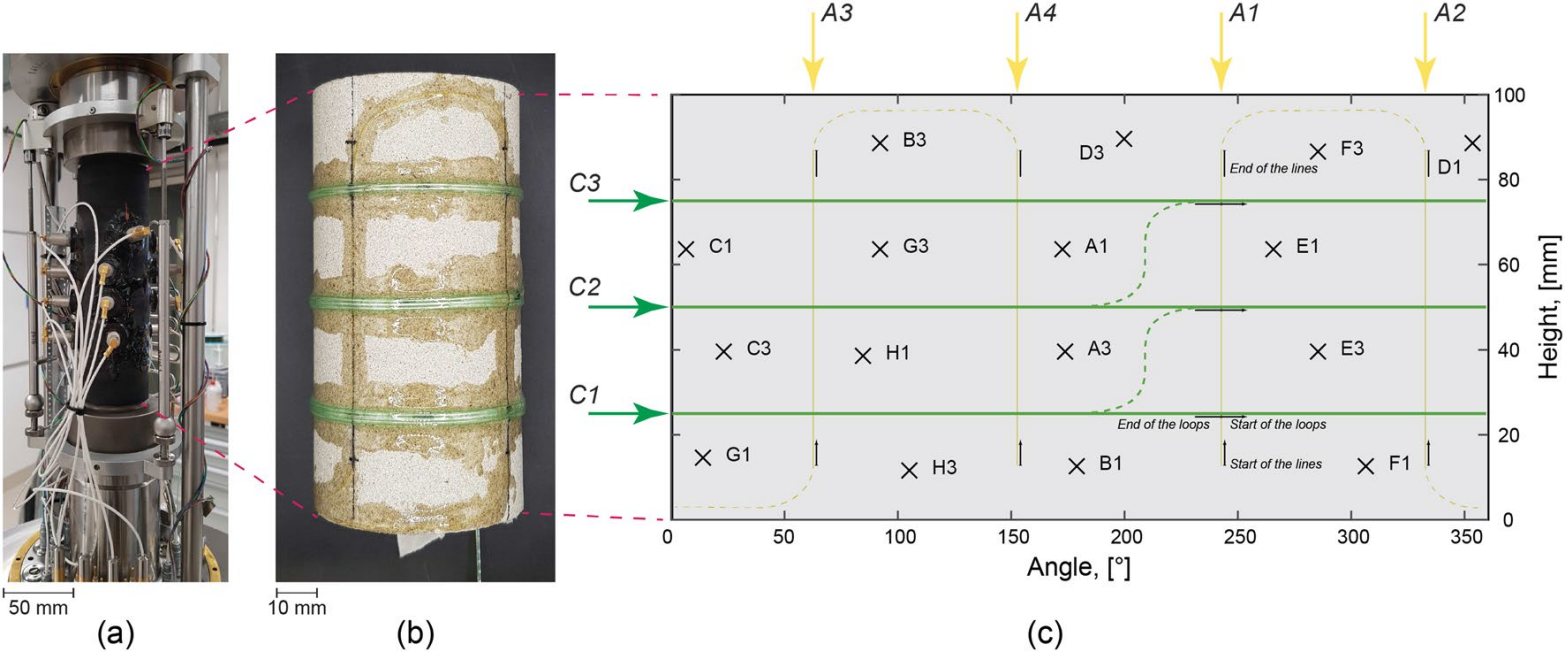
**a** Experiment assembly. **b** Berea sandstone sample with optical fibers glued on its surface. **c** Projection of the PZT (black crosses) and DSS (acrylate fiber in green and polymide fiber in yellow) sensor positions on the sample surface with respective channel names of the AE DAQ. *A1–A4* and *C1–C3* indicate the DSS lines and loops, respectively

The experiment was performed on a dry intact cylindrical sample of Berea sandstone (Fig. 1b). This was chosen because of its grain homogeneity and its well-known material properties resulting from past extensive, laboratory studies – making it a practical lithology for numerical comparisons. The sample weighed 436.70 g with a density of approximately 2120 kg/m$^3$, a height of 101.1 mm and a diameter of 50.9 mm. Berea sandstone is a well-sorted and fine-grained sandstone and is mainly composed by quartz with sparse amounts of feldspars, kaolinite and carbonate minerals (Lo et al. 1986). The permeability of Berea sandstone is typically in the range between 10$^{-14}$ and 10$^{-16}$ m$^2$ and the porosity is approximately 20% (Zoback and Byerlee 1975; Churcher et al. 1991; Selvadurai and Suvorov 2022). The rock sample was jacketed in a nitrile rubber (NBR) jacket as displayed in Fig. 1a.

### Laboratory Facility and Monitoring Sensors Employed

The experiment was conducted at the Rock Physics and Mechanics Laboratory (RPMLab) at ETH Zurich (Switzerland) using a triaxial rock testing apparatus (LabQuake). LabQuake is equipped with sensors that allowed us to monitor and study both fast (PZTs) and slow (DSS with optical fibers) deformation occurring throughout the experiment.

#### Acoustic Emission Monitoring System

Acoustic emissions were detected by employing 16 in-house developed conical-type PZTs, which were designed to resist high pressures and temperatures (Selvadurai et al. 2022). To install the PZTs in contact with the sample surface, the jacket was punched to create portholes for all of them (Fig. 1a). To avoid leakages of the confining oil into the jacket, we applied a double coating of epoxy (LOCTITE®EA 9455) around the portholes on the external side of the jacket. Throughout the whole experiment we continuously, passively recorded AEs with a sampling frequency of 10 MHz using a data acquisition system (DAQ, TraNET EPC-TPCE, Elsys AG). The recorded signals were amplified by 40 dB. To retrieve an updated wave velocity model during the experiment, this DAQ was also used to perform active surveys using a high-voltage multiplexer unit (HVP, AE-HV-MUX, Elsys AG), which employed a Piezosystem Jena voltage amplifier for pulse generation (HVP 1000/200) (Selvadurai et al. 2022). Ultrasonic surveys were conducted every two minutes by using a high-voltage pulse of 350 V. A total of 51 ultrasonic surveys, each composed of 10 pulses from each of the 16 PZTs, was produced. A schematic representation of the PZT positions is shown in Fig. 1c.

#### Strain Monitoring Techniques

Distributed strain sensing was deployed to measure strain on the sample surface by gluing the fiber optic cables with a two part epoxy directly on its outer surface and by punching an additional porthole in the jacket to allow the two optical fiber cables to exit it (details provided in Salazar Vásquez et al. 2022a). Two types of fiber optic cable were used: polymide and acrylate variants (provided by Iridis Solutions GmbH). The polymide fiber cable was used to measure axial strain $\varepsilon _{1}$, whereas the acrylate cable measured circumferential strain $\varepsilon _{3}$. The DSS data was acquired by employing a Luna OBR 4600 Optical Backscatter Reflectometer with a sampling frequency of 0.1 Hz and a spatial resolution along the fibers of 5 mm. The glued fibers formed four vertical lines (*A1–A4*) with the polymide fiber and three loops (*C1–C3*) with the acrylate fiber (Figs. 1b, c). We followed typical convention for rock mechanics, with positive compression and negative extension.

### Experimental Protocol

**Fig. 2:**
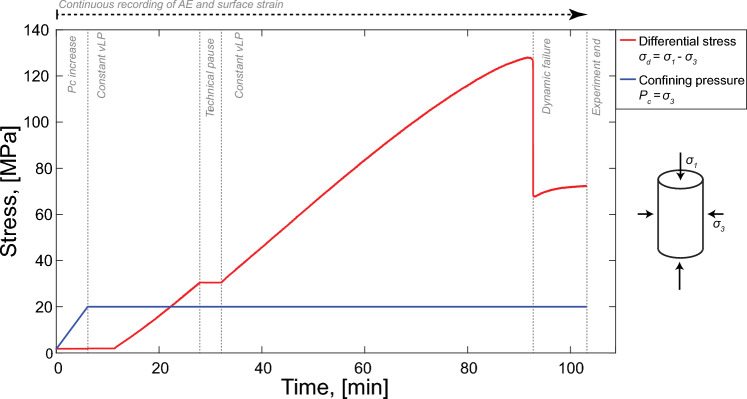
Protocol followed throughout the experiment. Differential stress $\sigma _d$ (red) and confining pressure $P_c$ (blue) as a function of time

The failure test was conducted following the protocol shown in Fig. 2, which was designed by consulting seminal studies of triaxial experiments on samples of Berea sandstone (e.g., Bernabe and Brace 1990; Menéndez et al. 1996). The sample was confined at 20 MPa, with a linear increase in confining pressure $P_c = \sigma _3$ equal to 3 MPa/min (i.e., confining stage). The differential stress $\sigma _d = \sigma _1-\sigma _3$ was subsequently increased by lowering the piston with a constant velocity v$_{LP}$ = 0.33 $\mu$m/s until the main failure of the rock sample, which resulted in a large $\sim$60 MPa stress drop (i.e., stage of differential stress increase). Loading was continued after failure for approximately 10 min. A technical pause in loading was performed at approximately 30 MPa differential stress to check the proper functioning of the AE and DSS data acquisition systems.

### Acoustic Emission Analysis

In the next sections we present the method followed (adapted from Bianchi et al. 2022) to analyse the large amount (2.1 Terabyte) of the single, continuous AE data file retrieved during the experiment but we first summarize the main steps performed. To be able to analyze the AE data, the single, continuous data file containing all the measurements retrieved by the 16 PZTs was parsed in shorter, sequential time windows. Two picking algorithms were run on the parsed files to determine the onsets of the AE waves and of the active pulses. By using the latter we constructed a time-varying homogeneous P-wave velocity model, which was further employed to locate the AEs. Additionally to the information presented in the next sections, we provide more details of the AE analysis workflow in the Appendix A.

#### Acoustic Emission Data Reduction

In order to analyze individual AEs we applied two different picking algorithms to parsed sections of the continuous data that covered 20 s of the experiment. A short-time average/long-time average (STA/LTA) (Vanderkulk et al. 1965; Trnkoczy 2009) and then an Akaike information criterion (AIC) (Akaike 1974; Kurz et al. 2005) algorithms were used to detect AEs within the continuous recording.

The STA/LTA algorithm was run on each of the 16 channels singularly and it individuated passive detections. Event classification was determined using the maximum time delay between detections on each channel and is discussed in more details in Appendix A. Acoustic emissions that were classified were then re-evaluated with the AIC picking algorithm but, before re-evaluating these, we applied a digital Butterworth bandpass filter (250–750 kHz) to the waveforms to increase the signal-to-noise ratio. We evaluated the AIC in the classified events and if the picks were too sparsely separated in time these were discarded. To further increase the picking accuracy, the accepted events were again re-evaluated with the AIC algorithm run on the raw waveforms and the accepted events that fitted the criteria were used to develop the passive AE catalogue.

#### Time-Varying Homogeneous P-Wave Velocity Model

Each survey consisted of 10 pulses on each sensor. We developed algorithms to isolate and stack these results (more details in Appendix A), where the time delay and distance between the sources and receivers were used to determine the velocity. The velocity values belonging to the same survey were finally averaged and, as a consequence, we were able to compute a time-varying homogeneous P-wave velocity model (see Appendix A, Fig. 12) consisting of 51 complete homogeneous velocity models, which were used to determine the locations of the AEs.

#### Acoustic Emission Localization

The AE catalogue was split into 51 consecutive, temporal sequences centred around the ultrasonic survey times. This allowed us to localize each AE with the most updated velocity model. To determine the AE locations we minimized the $L^2$-norm of a cost function *F* defined as follows:

1 $$||F(x_0,y_0,z_0,t_0) ||_{\min }^2=\sqrt{(x_0-x_s)^2+(y_0-y_s)^2+(z_0-z_s)^2} - v \cdot (t_{arr} - t_0)$$

with $(x_0,y_0,z_0)$ and $t_0$ being the source location coordinates and nucleation time, respectively, $(x_s,y_s,z_s)$ are the PZT coordinates, $t_{arr}$ the wave arrivals at the PZT locations and *v* the time-dependent velocity described in the previous section. After determining their locations, we selected only the AEs that were located with an accuracy of at least ± 2.5 mm inside the sample—a threshold value arbitrarily chosen to satisfy our requirements.

### Numerical Simulation Methodology

In this section we outline the computational modeling approach used in this study and describe the numerical setup and boundary conditions imposed during the simulations. The parameters chosen are provided in the Appendix B with explanations regarding the assumptions taken.

The simulations were conducted using the computational model Hydro-Mechanical Earthquake Cycles (H-MEC, Dal Zilio et al. 2022). Originally designed to explore solid–fluid interactions in subduction zones (Dal Zilio and Gerya 2022), H-MEC is a two-dimensional, continuum-based model that fully integrates poro-visco-elasto-plastic dynamics. This model distinguishes itself by its capability to analyze the evolution of elastic, viscous, and plastic strains, either in bulk rock volumes or along pre-existing faults. Such an approach has proven effective in investigating preparatory processes and strain localization at various scales—from large, subduction-related phenomena (Petrini et al. 2020; Dal Zilio et al. 2022; Dal Zilio and Gerya 2022) to reservoir (Petrini 2019; Bianchi 2020) and laboratory scales (Bianchi et al. 2022). Given its proven versatility and the specific challenges of modeling high-porosity lithologies like Berea sandstone, we enhanced H-MEC by incorporating a second phase to represent air within the porous matrix. This modification made H-MEC an ideal choice for capturing the intricate preparatory processes we aimed to study.

#### Numerical Approach and Governing Equations

H-MEC is a two-dimensional continuum-based and fully coupled seismo-hydro-mechanical poro-visco-elasto-plastic numerical approach based on a rheological formulation constituted by the compressible linear Maxwell model for viscoelasticity and the nonassociated Drucker–Prager plasticity model (Prager and Drucker 1952; Gerya 2019). It incorporates a staggered finite difference marker-in-cell method, Picard iterations and adaptive time stepping (Gerya 2019; Gerya and Yuen 2007; Petrini et al. 2020; Dal Zilio et al. 2022). The governing equations contain poroelasticity terms consistent with Biot’s theory (Biot 1941; Gassmann 1951; Yarushina and Podladchikov 2015), including the Biot–Willis coefficient (Biot and Willis 1957) and the Skempton coefficient (Skempton 1960; Bishop 1973), which allow for a fully coupled pressure-based compressible formulation. This hydro-mechanical system thus accounts for the compressibility of both the solid matrix and fluid phase through the elastic (reversible), visco-plastic (irreversible) (de)compaction and plastic dilation of the interconnected porous space. The numerical solver finds a unique solution by simultaneously solving four main governing equations: the total momentum, the fluid momentum, the solid mass and the fluid mass conservation equations, respectively (Yarushina and Podladchikov 2015; Gerya 2019; Petrini et al. 2020; Dal Zilio et al. 2022):

2 $$\begin{aligned}{} & {} \triangledown \cdot \sigma + \rho _t \textit{g} = \rho _t \frac{D^s \textit{v}^s}{Dt} \end{aligned}$$

3 $$\begin{aligned}{} & {} \textit{v}^D = - \frac{k}{\eta _f} \Big (\triangledown p_f - \rho _f \textit{g} + \rho _f \frac{D^s \textit{v}^s}{Dt}\Big ) \end{aligned}$$

4 $$\begin{aligned}{} & {} \triangledown \cdot \textit{v}^s = - \frac{1}{K_d}\Big ( \frac{D^s p_t}{D_t} - \alpha \frac{D^f p_f}{D_t} \Big ) - \frac{p_t - p_f}{\eta _\phi (1-\phi )}\nonumber \\ {}{} & {} \qquad \qquad + 2\cdot \sin (\psi ){\dot{\varepsilon }}_{II(plastic)} \end{aligned}$$

5 $$\begin{aligned}{} & {} \triangledown \cdot \textit{v}^D = \frac{\alpha }{K_d} \Big ( \frac{D^s p_t}{Dt} - \frac{1}{\beta } \frac{D^f p_f}{Dt} \Big ) + \frac{p_t - p_f}{\eta _\phi (1-\phi )}\nonumber \\ {}{} & {} \qquad \qquad - 2\cdot \sin (\psi ){\dot{\varepsilon }}_{II(plastic)} \end{aligned}$$

where $\sigma$ is the total stress tensor, $\textit{v}^s$ and $\textit{v}^D$ respectively are the solid and Darcy velocities, $p_t$ and $p_f$ respectively are the total and fluid pressures, *g* is the gravitational acceleration, $\rho _t$ and $\rho _f$ respectively are the total (bulk) and fluid densities, *k* is the permeability, $\eta _f$ is the fluid dynamic viscosity, $K_d$ is the drained bulk modulus, $\alpha$ and $\beta$ respectively are the Biot–Willis and Skempton coefficients, $\phi$ is the porosity, $\eta _\phi$ is the effective visco-plastic compaction viscosity, $\frac{D^s(\cdot )}{Dt}$ and $\frac{D^f(\cdot )}{Dt}$ respectively are the Lagrangian time derivative in the solid and fluid reference frame and *t* is the time. For further details related to the numerical solver definition, we refer the reader to Gerya (2019).

With respect to the mathematical formulation presented in Dal Zilio et al. (2022), we adapted H-MEC in response of the physical requirements of our laboratory setup. We added a plastic term $\Gamma _{plastic}$ that considers volumetric changes during plastic deformation due to rock dilation in the solid (positive term, Eq. 4) and fluid (negative term, Eq. 5) mass conservation equations as formulated by Gerya (2019) (Eq. 12.63):

6 $$\begin{aligned} \Gamma _{plastic} = 2\cdot \sin (\psi ){\dot{\varepsilon }}_{II(plastic)} \end{aligned}$$

with $\psi$ being the dilation angle and ${\dot{\varepsilon }}_{II(plastic)}$ the second invariant of the deviatoric plastic strain rate tensor that is defined as ${\dot{\varepsilon }}_{II(plastic)} = \frac{\sigma _{II}}{2} \Big (\frac{1}{\eta _{vp}} - \frac{1}{\eta }\Big )$ with $\eta _{vp}$ and $\eta$ respectively being the effective visco-plastic and ductile viscosities (Gerya 2019). $\sigma _{II}$ is the second invariant of the deviatoric stress tensor and is defined as $\sigma _{II} = \sqrt{{\textstyle \frac{1}{2}}\sigma ^{_{'2}}_{ij}}$ with the subscripts *ij* implying Einstein notation (Gerya 2019).

The terms $\zeta _{elastic} = \frac{1}{K_d}\Big ( \frac{D^s p_t}{Dt} - \alpha \frac{D^f p_f}{Dt} \Big )$, $\zeta _{viscoplastic} = \frac{p_t - p_f}{\eta _\phi (1-\phi )}$ and $\Gamma _{plastic}$ (Eq. 6) are present in both solid (Eq. 4) and fluid (Eq. 5) conservation equations and are strain rates, which quantify the reversible elastic (de)compaction (Dal Zilio et al. 2022, Eq. 50), viscoplastic compaction (Dal Zilio et al. 2022, Eq. 49) and dilation of the pore space, respectively. These parameters are crucial in the simulations of such laboratory tests, because they quantify the volumetric deformation of the sample. These terms are also found in the fluid mass conservation equation (Eq. 5), since the solver fully couples the solid and fluid phases.

The dissipation of mechanical energy *D* is a term related to shear heating caused during irreversible plastic deformation and is a suited parameter to track seismic (i.e., AEs) or aseismic strain occurring within the sample. The dissipation can be calculated with the deviatoric stresses $\sigma _{ij}^{'}$ and strain rates ${\dot{\varepsilon }}_{ij}^{'}$ as Gerya (2019):

7 $$\begin{aligned} D = \sigma ^{'}_{ij}\cdot {\dot{\varepsilon }}^{'}_{ij} \end{aligned}$$

Due the brittle nature of the deformation occurring during triaxial tests performed on sample of Berea sandstone at similar confining pressures as performed in this test (e.g., Menéndez et al. 1996), we will assume contributions in the simulated processes from viscosity-related terms to be negligible.

#### Numerical Model and Boundary Conditions

**Fig. 3:**
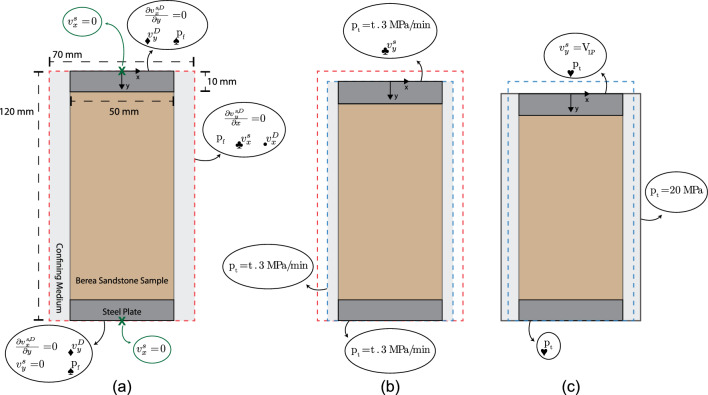
**a** Initial two-dimensional model setup including the rock sample, two steel plates at the top and bottom boundaries and the confining medium. The boundary conditions displayed were employed throughout the entire simulation. The boundary conditions imposed at the right and left boundaries were always equal (i.e., symmetrical with respect to the y-axis). The green crosses indicate the locations where the model was fixed in the horizontal direction. **b**–**c** Model setup in the two stages of the simulation. The boundary conditions displayed in (**b**) and (**c**) were only valid in the specific stage. The sample dimensions are exaggerated for visualization purposes. The boundary conditions linked with a symbol were retrieved by solving the following equations: $_\bullet$x-fluid momentum (Eq. 3), ♦ y-fluid momentum (Eq. 3), ♥ solid continuity (Eq. 4), ♠ fluid continuity equations (Eq. 5) and ♣ Eq. 8

Simulations were performed using the setup shown in Fig. 3a, which was built in correspondence to the laboratory experiment design. The computational domain included a unique fluid phase, which was air in our dry test, and three solid phases representing the Berea sandstone sample, the steel loading plates, and the confining medium (assumed to be water for simplicity) in the triaxial cell (see Appendix B for parameter choice and motivation). The initial dimensions of the domain were 70 mm in width and 120 mm in height, with the sample and plates measuring 50 mm of the width and the confining medium imposed at both side of them (Fig. 3a). The mesh was defined by a 71 $\times$ 121 node grid, with initial horizontal and vertical spatial resolutions of 1 mm. At the end of every timestep, the domain dimensions and the spatial resolutions were adjusted according to the boundary conditions imposed (see Fig. 3 and caption for more details). To quantify the macroscopic deformation during the simulation, we tracked the relative distance between pairs of tracer markers placed on the sample surface both in the axial and circumferential directions. For comparison purposes we only used markers that were initially located at the same height on the sample (279 markers on each side with an initial relative distance of 0.25 mm) as the four axial lines during the test (*A1–A4*, Fig. 1c).

As in the laboratory test, the numerical simulations were divided into two distinct stages: an increase of confining pressure (Fig. 3b) and an increase of differential stress until failure of the sample was reached (Fig. 3c). During the confining stage, the total pressure $p_t$ was increased along the four domain boundaries with a constant gradient of 3 MPa/min until a total pressure $p_t$ of 20 MPa was achieved. Simultaneously, the perpendicular components of the solid velocity at the top and at the two lateral boundaries were adjusted so that the normal total stresses $\sigma$ at these boundaries corresponded to the total pressure $p_t$ value imposed (the normal deviatoric stresses $\sigma '$ were set to be equal to 0 Pa). These stress boundary conditions were mathematically formulated as Bianchi (2020), Gerya (2019):

8 $$\begin{aligned} \begin{aligned} \int \sigma{'}_{xx} \, dy = \int \left( 2\eta _{vp}\Big (\frac{2}{3}\frac{\partial v^s_x}{\partial x}-\frac{1}{3}\frac{\partial v^s_y}{\partial y}\Big )Z+\sigma ^{'0}_{xx}(1-Z)\right) \, dy = 0 \, \text {Pa} \\ \int \sigma{'}_{yy} \, dx = \int \left( 2\eta _{vp}\Big (\frac{2}{3}\frac{\partial v^s_y}{\partial y}-\frac{1}{3}\frac{\partial v^s_x}{\partial x}\Big )Z+\sigma '^{0}_{yy}(1-Z)\right) \, dx = 0 \, \text {Pa} \end{aligned} \end{aligned}$$

where $\sigma ^{'0}$ is the deviatoric stress of the previous time step and Z is a visco-elasticity term (Petrini et al. 2020, Eq. 26). This boundary condition implied that a unique value of solid velocity $v^s$ was imposed. The dimensions of the numerical domain were updated accordingly with the velocities imposed and the timestep length at the end of each iteration. The model conserved the number of nodes in the numerical domain while the horizontal and vertical mesh resolutions were updated accordingly.

As a total pressure $p_t$ = 20 MPa (i.e., the confining pressure applied in the experiment) was reached along the four boundaries, boundary conditions were modified to mimic the axial loading of the sample. For this, at the two lateral boundaries, a constant total pressure $p_t$ = 20 MPa was imposed, along with stress boundary conditions as previously presented in Eq. (8). On the other hand, for the horizontal boundaries, the total pressure was imposed by solving the solid continuity equation (Eq. 4). At the top boundary, we imposed $v_y^s$ = 0.33 $\mu$m/s, matching the loading plate velocity $v_{LP}$ used during the failure test, which was kept constant during the entire simulation. The domain dimensions and spatial resolutions were adjusted as explained earlier.

During both phases of the simulations (Fig. 3a), the solid y-velocity of the bottom boundary was $v_y^s = 0$ m/s and fluid pressure $p_f$ was determined by solving the fluid continuity equation (Eq. 5) at the four boundaries. The parallel components to the boundaries of the solid $v^s$ and Darcy $v^D$ velocities were adjusted to avoid gradients across the boundaries ($\frac{\partial v^{s,D}_x}{\partial y} = \frac{\partial v^{s,D}_y}{\partial x} = 0$ m/s; free slip conditions). The only exceptions made were for the two single nodes in the middle of the domain (green crosses in Fig. 3) at the top and bottom boundaries, where we imposed $v_x^s = 0$ m/s. This choice was made to avoid lateral movements of the two plates and to stabilize the simulations. The Darcy x- and y-velocities normal to the four boundaries were determined by solving the x and y fluid momentum equations (Eq. 3) with fluid pressure $p_f$ equal to atmospheric pressure at the boundaries (Bianchi 2020). All internal boundaries (i.e., plate-sample, sample-confining medium and plate-confining medium) were governed by continuity conditions.

## Experimental Results

### Mechanical Data

**Fig. 4:**
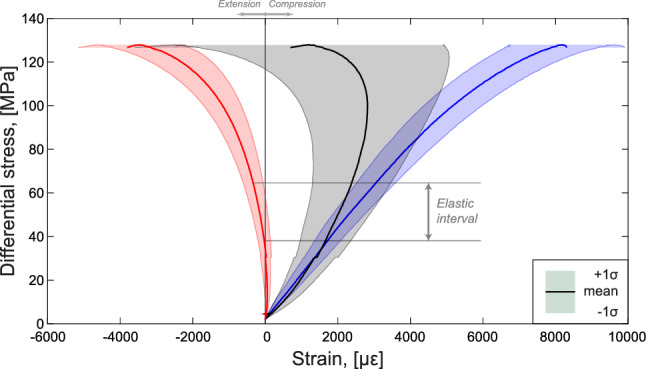
Stress–strain curves determined with the DSS measurements. Differential stress as a function of the axial (blue), circumferential (red) and volumetric (black) strains. The grey horizontal lines at 30–50% of the peak differential stress indicate the region over which the elastic moduli were calculated

During the experiment, a peak differential stress $\sigma _{peak}$ of approximately 128 MPa was reached (Figs. 2 and 4) and, shortly after it, a major stress drop of $\sim$60 MPa associated with catastrophic failure was observed. In Fig. 4 we show the stress–strain curves determined with measurements from the DSS data determined as the mean $\mu$ of all point measurements along the four vertical optical fiber lines and the three loops (see Fig. 1). The volumetric strain was computed as $\varepsilon _{v} = \varepsilon _{1} + 2 \cdot \varepsilon _3$ from the axial and circumferential DSS data shown in Fig. 4. The shaded areas were built using the standard deviation $\sigma$ of the DSS measurements with the limits being $\mu \pm \sigma$. The dimensions of these areas are representative of strain localization, as an increase of these reflects an increase of the difference of deformation measurements we were retrieving during the experiment.

We calculated the elastic properties (Young’s modulus and Poisson’s ratio) in the interval highlighted with horizontal black lines in Fig. 4 that was determined by the 30–50% of the peak differential stress (Bieniawski and Bernede 1979). The Young’s modulus determined from the averaged axial surface strain data equaled approximately *E* = 20 GPa. The Poisson’s ratio was calculated by taking the ratio of the averaged circumferential to averaged axial DSS strains in the same elastic interval and was found to be approximately $\nu$ = 0.23. These values of Young’s modulus and Poisson’s ratio were used as inputs to the simulation. The standard deviations showed to increase with increasing differential stress. We note that the sample underwent volumetric contraction until $\sim$100 MPa where it then experienced relative dilation. This behavior is generally understood as the development of a shear fracture (e.g., Jaeger et al. 2007; Paterson and Wong 2005). Dilation continued until failure, where the optical fibers were broken and no more DSS data was available.

### Observations of Seismic Clustering

**Fig. 5:**
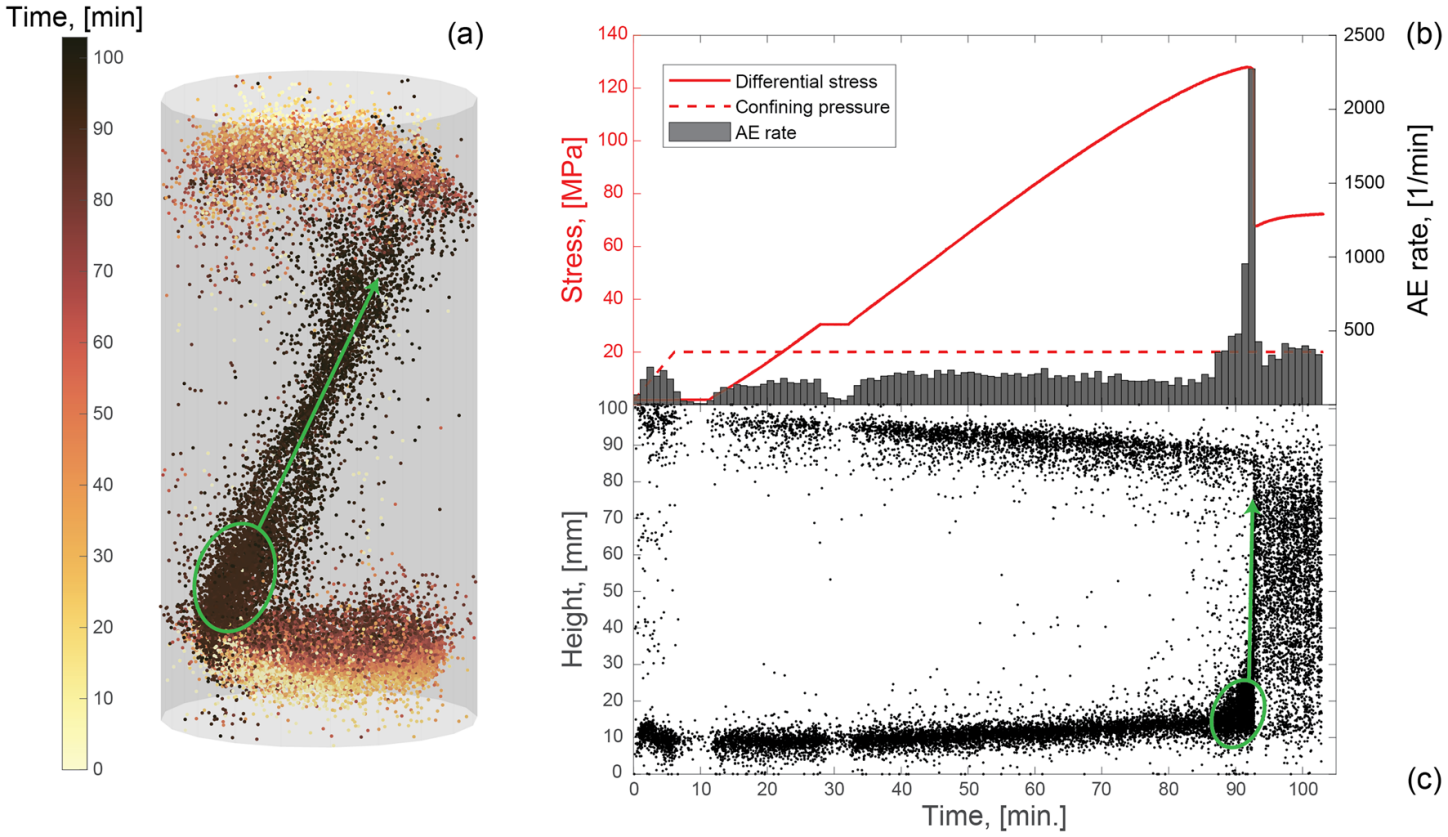
**a** AE locations colored with respect to the time from the start of the experiment. **b** AE rate, differential stress and confining pressure as a function of time. **c** AE height positions as a function of time. The green circles and arrows highlight the AE localization region in the second regime and the propagation direction of the main fracture, respectively

During the test, 22,511 AEs were localized. Figures 5a, c show the AE source locations as a function of time, whereas Fig. 5b displays the AE rate, the differential stress and the confining pressure, also as a function of time. By combining observations linked to the AE locations and rate, it was possible to individuate three different seismic regimes throughout the test. The first regime consists of AEs that concentrated into two distinct clusters at the top and bottom of the sample, with significantly more AEs counted in the latter one. During this regime, the AE rates oscillated between $\sim$100–250 AEs per minute. The second regime was characterized by an increase of the AE rate corresponding to an increased amount of AE sources localized on one side of the lower half of the sample (green circles in Figs. 5a, c). Shortly ($\sim$2 min) before the main failure, the AE rate spiked reaching a maximum value of approximately 2300 AEs/min before decreasing immediately after the stress drop to a rate smaller than 500 AEs/min. The second regime was also associated with the development of the main fracture, which propagated sub-vertically from one side of the lower half of the sample to the opposite side of the upper half (green arrows in Figs. 5a, c highlight the propagation direction). In the last regime, the AEs were localized along or in the proximity of the nucleated macrofracture, highlighting its relative position in the sample. In this regime, the AE rate was higher than in the first regime, fluctuating by about $\sim$400 AEs per minute.

### Spatio-Temporal Variation of Strain

**Fig. 6:**
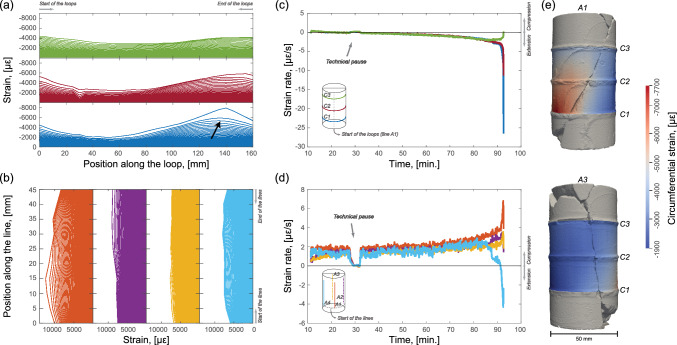
**a** Circumferential and **b** axial distributed surface strain measurements with increasing time (each line corresponds to a time measurement along the optical fibers). The measurements were offset to the start of the differential stress increase. The black arrow in (**a**) indicates an extensional peak. **c** Circumferential and **d** axial strain rates as a function of time calculated as the average of each loop or line, respectively. **e** Post-mortem scan at 2 different angles (0–180$^\circ$) of rotation of the rock sample with interpolated circumferential strain field superimposed. The circumferential strain field belongs to the last measurement retrieved from the three DSS loops prior to the macroscopic failure of the sample

The circumferential and axial DSS measurements retrieved during the test detected localization of strain prior to the sample failure. Figures 6a, b display temporal isochrones of the circumferential and axial distributed surface strain measurements, respectively; for visualization purposes, each line represents only one in every ten measurements taken. The temporal isochrones are offset to the start of the differential loading phase (see Fig. 2) and are shown until the last measurement available prior to failure. In Fig. 6a we see that the *C1* loop, located in the bottom half of the sample, exhibits an extensional peak (black arrow) that was associated with strain localization near this region of the sample. This relative extensional regime can also be followed in the last fourth (between lines *A4* and *A1*) of the loops *C2* and *C3*, although the absolute values were lower with respect to *C1*. Figure 6b shows the distributed axial measurements that did not generally show strain concentrations on the sample surface. Lines *A2*, *A3* and *A4* presented approximately homogeneous compressional strain distribution. In line *A1* some variation in strain was observed along the axial fiber, where the larger values corresponded to where the strain was concentrated in the circumferential line C2. In general, the axial strain values of line *A1* were higher than in the other three lines, indicating larger compression on that side of the sample.

Circumferential (Fig. 6c) and axial (Fig. 6d) strain rates were calculated by averaging the evolution of strain in time of each loop and line, respectively. The three loops showed to be similarly in extension up to $\sim$87 min in the test with strain rates slightly increasing up to approximately $-3\,\mu \varepsilon /\text {s}$. Within the last $\sim$5 min prior to the sample failure, loops *C1* and *C2* experienced an abrupt acceleration of extension with the strain rates reaching approximately $-27$ and $-12\,\mu \varepsilon /\text {s}$, respectively. During the same period, *C3* showed to decrease its extensional trend back to $\sim$0 $\mu \varepsilon /\text {s}$. Each line showed measurements indicative for a slightly increasing compression with strain rates in the range $\sim$2–3 $\mu \varepsilon /\text {s}$ up to $\sim$87 min. After that the axial lines also detected an acceleration process, even though the strain rates did not grow to similar levels as the three loops. The strain rates of lines *A1*–*A3* increased up to the pre-last measurement available before falling back to lower values ($\sim$2–4 $\mu \varepsilon /\text {s}$) before the fracture nucleation. Line *A4* directly moved towards a dilation regime and the strain rate accelerated to approximately $-4\,\mu \varepsilon /\text {s}$.

The sample was scanned post-mortem with a high resolution 3D optical scanner (Artec Space Spider) that is based on blue light technology (Fig. 6e). We linearly interpolated the circumferential strain field from the last measurement taken before macroscopic failure of the three DSS loops and we superimposed it on the scan (Salazar Vásquez et al. 2022a). As shown in Figs. 6a, b, the higher magnitude of circumferential strain was localized in the proximity of the end of the *C1* loop and bottom of the *A1* line. This spatially correlates with the relative surface position of the macroscopic fracture in the sample before the dynamic stress drop. This is further supported by the AE locations in the second regime (see green circles in Figs. 5a, c). The observations presented showed that strain mainly localized in the lower part of the sample prior to the main failure and this localization could be recognized by both the distributed strain measurements and by an acceleration of the strain rates.

### Ultrasonic Surveys

We tracked P-wave velocity changes determined using active surveys performed every two minutes during the test. With this we reconstructed an average time-varying homogeneous P-wave velocity model (Appendix A, Fig. 12) that was employed to locate the AEs (Eq. 1). During the confining stage, we noted an increased P-wave velocity from approximately 3100 m/s to almost 3400 m/s. Additionally, we observed that during the loading stage, the velocity was further raised to over 3400 m/s. However, as the test approached the main failure, it strongly decreased, reaching $\sim$ 3000 m/s at the last survey prior to it. A similar behaviour was observed in past studies on Berea sandstone samples (e.g., Scott et al. 1993).

**Fig. 7:**
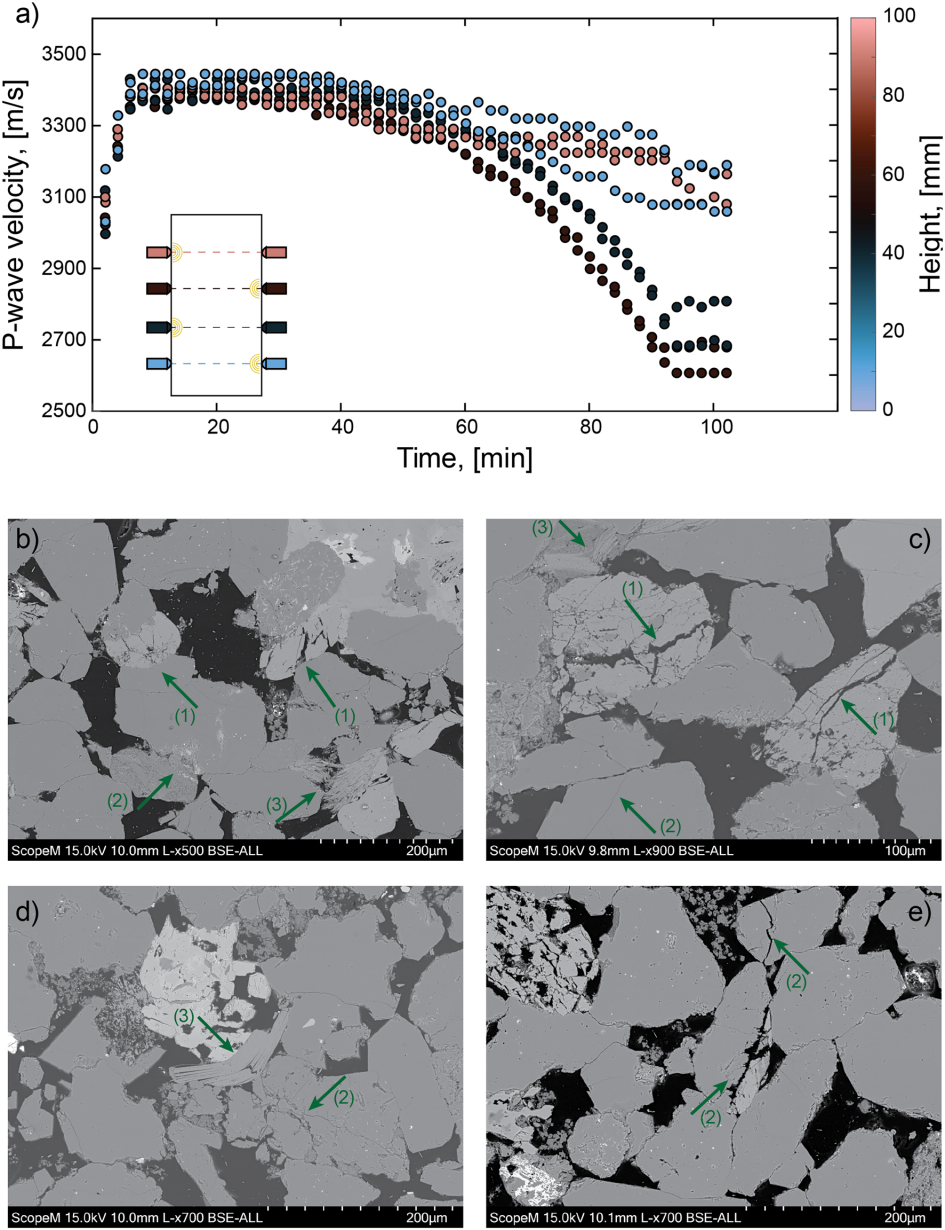
**a** Time-varying homogeneous P-wave velocity models of different sensor pairs colored by the sensor height. **b**–**e** Backscattered SEM micrographs of thin sections retrieved in central regions of the sample. The green arrows indicate points of interest in which grains of (1) feldspar, (2) quartz or (3) clay minerals were damaged, deformed or displaced

The standard deviation of the P-wave velocities increased throughout the test; this is shown in Fig. 7a. The velocity models of the single sensor-receiver pairs across the top, middle and bottom of the sample, showed a common trend up to approximately 50 min from the start of the test. After this the velocity models built with the sensor pairs located at the top and bottom of the sample did not show a strong decrease ($\sim$200–300 m/s) when approaching sample failure. On the other hand, the velocity calculated from the two lines of sensor pairs situated towards the middle of the sample showed a decrease that reached values in the range 2600–2700 m/s. These variations in velocity evolution between sensor pairs towards the ends and the middle of the sample in the second half of the experiment were the cause for the increase of the standard deviations of the average time-varying homogeneous P-wave velocity model shown in Appendix A and might highlight a preparatory process that occurred aseismically within central regions of the specimen. To support this hypothesis, we performed a microscopical analysis on thin sections retrieved from these central regions but away from the macrofracture. We employed a backscattered scanning electron microscope (SEM, Hitachi—SU5000) and we displayed the results within four micrographs (Figs. 7b–e). The green arrows indicate points of interest of the sections in which we observed grains of feldspar, quartz or clay minerals to be significantly damaged (inter- or intragranular cracking), deformed or displaced (e.g., Baud et al. 2004; Fortin et al. 2006). Even though the sections were retrieved from a considerable distance from the macrofracture, we can not exclude the fact that the dynamic rupture occurred at the end of the experiment might have induced additional overprinted damage.

## Numerical Results

### Macroscopic Deformation

**Fig. 8:**
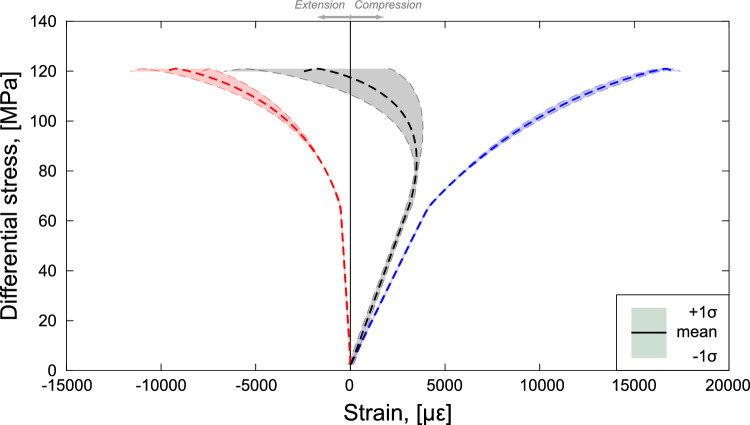
Stress–strain curves determined with the numerical simulations. Differential stress as a function of the axial (blue), circumferential (red) and volumetric (black) strains

Similar to the approach taken with the laboratory DSS measurements, we determined the mean ($\mu$) axial $\varepsilon _1$, circumferential $\varepsilon _3$ and volumetric strains ($\varepsilon _v = \varepsilon _1 + 2 \cdot \varepsilon _3$) by averaging the relative displacement between all the marker pairs (Fig. 8). The shaded areas depicted in Fig. 8 reflect the standard deviation ($\mu \pm \sigma$) of the deformation measurements retrieved during the simulations and were determined in accordance with the laboratory results (Fig. 4).

After confining the sample, the upper boundary moved downwards and the sample responded elastically. This caused the axial strain to rise linearly in compression while the circumferential strain decreased proportionally in extension. At $\sigma _d\sim 65$ MPa the deformation transitioned from a purely elastic state to a yielding regime. A peak stress of approximately 120 MPa was reached before the failure. As H-MEC could not capture the dynamic stress drop linked to the nucleation of the macrofracture, we only presented results up to that moment. The macroscopic deformation matched, at a first-order, the experimental observations; however, some limitations of the numerical model were certainly present and should not be neglected. The numerical axial strain showed a stronger compressional behaviour with respect to the DSS data, whereas the circumferential strain appeared to dilate more compared to the DSS results. Discrepancies regarding the onset of dilatancy were also remarked between the simulated and experimental data. Relative dilatancy was observed at a differential stress of approximately 100 MPa during the test. However, this onset was recorded at a lower differential stress during the simulations ($\sim$80–85 MPa). As observed in the laboratory test (Fig. 4), the standard deviations of the strain measurements increased with increasing differential stress (Fig. 8).

### The Evolution of the Dissipation of Mechanical Energy

**Fig. 9:**
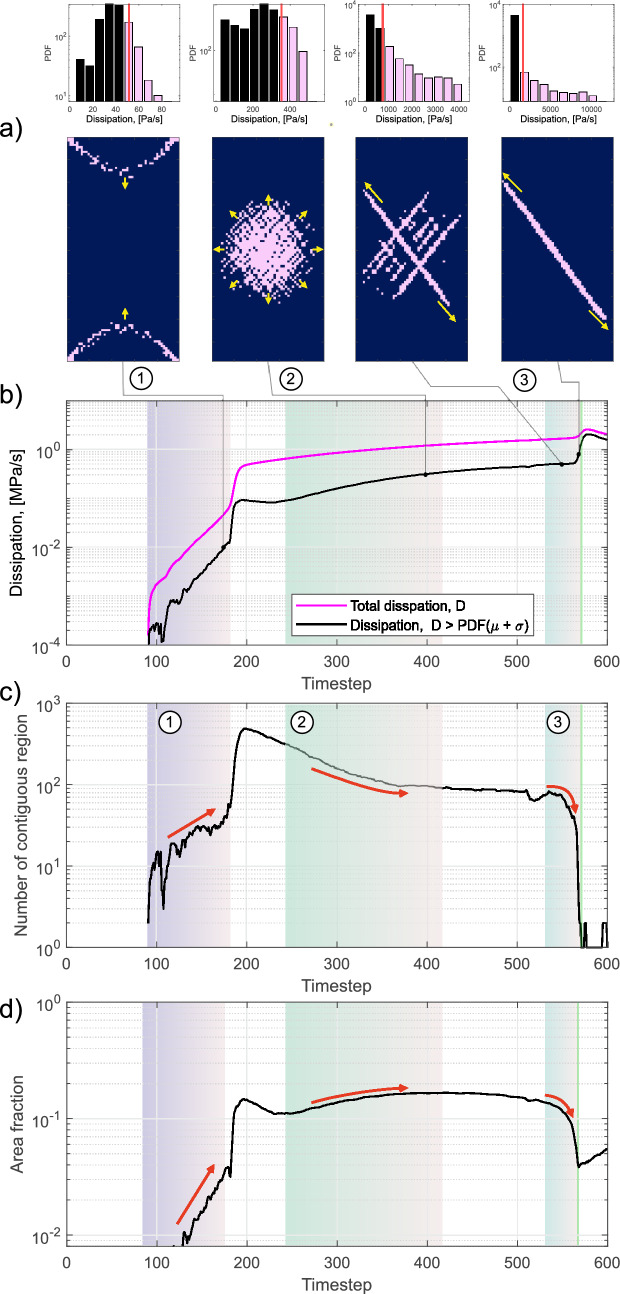
Numerical results showing the spatial–temporal evolution of regions experiencing higher levels of dissipation. **a** A binary mask was used to isolate a ROI in the model experiencing levels of dissipation $D \ge \mu + \sigma$ as determined from the PDF at each timestep. Four snapshots in time are shown with their PDF and the mask applied to show the ROI composed of highly dissipative elements (pink regions). Three stages related to localization of these dissipative elements were described in the text. **b** Comparison of the total dissipation (magenta) to the sum of dissipation in ROI (black). Using an image detection algorithm, we were able to track **c** the number of contiguous regions and **d** the area fraction within the model. This provided us with insights into the localization of dissipation, which is a proxy for strain rate

In Fig. 9 we investigated the spatial distribution of the irreversible dissipation *D* (Eq. 7). The mechanisms contributing to the dissipative processes were not examined in this study; however, we studied regions in the model that were experiencing higher level of dissipation throughout the experiment. We employed image detection algorithms to track the clusters and number of elements that were experiencing the higher level of dissipation and we noted that these regions displayed prominent localization in the last moments leading up to the sample failure.

Figure 9a shows images of the numerical results at four timesteps $t_{step}$ = 175, 400, 561 and 571. In each timestep, we calculated the dissipation at each element and represented them as a probability distribution function (PDF) as shown in the panels above for reference. To isolate only the elements experiencing higher levels of dissipation, the mean $\mu$ and standard deviation $\sigma$ were calculated. We chose to isolate element where $D \ge \mu + \sigma$ (i.e., mask generation), which were shown as the pink values of the PDF and the pink elements in the spatial distribution (lower panels of Fig. 9a). Regions sustaining higher levels of dissipation *D* showed clear patterns in their spatial distribution throughout the simulated test.

We compared the total dissipation to regions sustaining higher level of dissipation through the simulation in Fig. 9b. While the regions experiencing higher levels of dissipation represented only a fraction of the total dissipation throughout the simulation, these appeared to localize and dictated important processes related to the nucleation and generation of the final macrofracture. We employed the image detection algorithm *regionprops* (MATLAB) to track two important metrics by exploiting the mask of regions experiencing high-levels of *D*: (i) the number of contiguous regions (Fig. 9c) and (ii) the total area fraction (Fig. 9d). The area fraction is the sum of all elements (i.e., grid nodes) with higher dissipation divided by the total number of elements of the sample ($98 \times 49 = 4802$). These two metrics were sufficient to allow us to discuss the localization of regions that were sustaining high levels of dissipation, which is a proxy for region experiencing higher strain rates (as per the definition before).

In Fig. 9, we used the high dissipation regions to highlight three stages in the simulation. Stage 1 consisted of high dissipation front that propagated away from the top and bottom of the sample, as denoted by the yellow arrows, when the differential stress was applied. This occurred between $t_{step}\sim$91 to 182. We note that both the number of contiguous region and area fraction of highly dissipative elements increased in this stage as denoted by the red arrows in Figs. 9c, d. Following this stage, there was a brief interval of diffuse dissipation throughout the sample, eventually settling in a region at the center of the sample that began to exhibit a higher level of dissipation. During stage 2 ($t_{step}\sim$244 to 425), we observed a decrease in the number of contiguous regions, accompanied by an increase in the area fraction. As shown schematically in Fig. 9a, we could observe that the region of high *D* grouped in the middle and spread outwards (yellow arrows) becoming larger in area and in consistency. This trend continued until dissipative conjugate bands began to form at the start of stage 3 ($t_{step}\sim$537). As these bands formed, they dominated the dissipation within the system and coalesced both spatially and temporally. This could be clearly observed by the sharp drop in number of contiguous region to a single region and also by a decrease in the area fraction that sustained the high-levels of dissipation. The formation of this single dissipative band is indicative of localization occurring within the model. The green line in Figs. 9b–d indicates the moment when the dissipation band reached the edge of the sample. At this point, the model produced a non-physical response due to its inability of dynamically solving the stress drop and relative fracture propagation. This resulted in a dissipation drop as the top hanging wall simply “flowed” across the foot wall over the shear zone that was produced during the localization of high dissipation regions. However, the angle of the shear zone formed during the simulation was $\sim$33 $^\circ$, which, at a first-order approximation, matched the angle estimated from the AE locations during the test ($\sim$30$^\circ$).

## Discussion

### Increased Seismic Activity due to Strain Localization

**Fig. 10:**
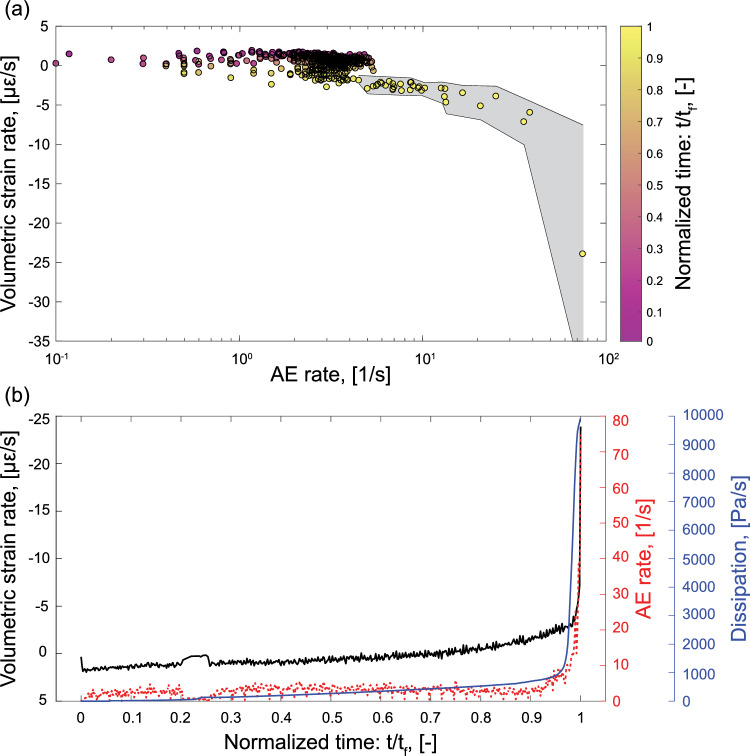
**a** Mean volumetric strain rate as a function of the AE rate from the start of the differential stress increase up to the last available DSS measurements. The colorbar indicates the normalized time to failure $t/t_f$ and the shaded gray area represents the $\pm \sigma$ of the volumetric strain rate as the standard deviation started to consistently grow before failure. **b** Comparison of the mean volumetric strain rate (black), AE rate (red) and dissipation (blue) as a function of the normalized time to failure $t/t_f$. The dissipation was retrieved during the numerical simulations, whereas both mean volumetric strain and AE rates during the laboratory test

In Sect. 3.3, we highlighted that the experimental findings, which show regions of the sample experiencing higher extensional circumferential strain (Figs. 6a, e) or strain rate (Fig. 6c) just prior to failure, qualitatively match the position of the AE clusters that precede the nucleation of the macrofracture. However, this was only representative for the last moments prior to the impending stress drop. To investigate whether the occurrence of AEs and deformation in the sample were linked during the test, we showed the mean volumetric strain rate in relation to the AE rate in Fig. 10a. Time was normalized by the failure time $t_{f}$, which allowed for a more appropriate comparison with the numerical results. We note that during the majority of the test, the volumetric strain rate fluctuated within the range ±3 $\mu \varepsilon /\text {s}$, while the AE rate was $\sim$1–6 AE/s, with both rates remaining relatively constant. As the test proceeded towards failure (i.e., $t/t_{f} \rightarrow 1$), both the volumetric strain and AE rates began to increase in unison. While Fig. 10a does not allow for a spatial understanding, we know this transition was likely related to failure initiation that resulted in the increase of seismicity localized on one side of the sample (green circle in Figs. 5a, c) and was perhaps the consequence of the accelerated deformation that was also captured in this region by the DSS array. Furthermore, both rate increases could be associated with a common preparatory process and we used the numerical results to shed light on these observations.

The gray shaded area shown in Fig. 10a shows the standard deviation ($\pm \sigma$) of the volumetric strain rate determined from the DSS array in the latter stages of the failure sequence. We remark that the standard deviation increased and that this trend was representative for the dispersion in the strain rate, a feature that is a further indication of strain localization in the presence of a nucleating macrofracture. We note that detecting this increase in dispersion would have not been feasible with strain monitoring techniques that rely on single point measurements (e.g., linear variable differential transducers or strain gauges). Even more sophisticated methods, such as dynamic X-ray tomography (e.g., Renard et al. 2009, 2017, 2019, McBeck et al. 2021b, 2022a, b), offer increased spatial resolution but less temporal resolution during the acceleration phase of the failure sequence. Moreover, these experiments offer limited insight into the seismic and aseismic dissipation of energy but show that it is important to characterize both (Cartwright-Taylor et al. 2022).

Understanding the mechanisms leading to runaway rupture and brittle failure offer unique insight into the failure and faulting process. Current observations of failure sequence before large earthquakes have noted that slow/aseismic preparation processes may be widespread in regions that eventually culminate in the hypocenter of the mainshock (Bürgmann 2014; Obara and Kato 2016; Bletery and Nocquet 2023). In many cases, regions that host this widespread slow deformation were also recognized to have notable increase in precursory seismic activity (Kato and Nakagawa 2014; Kato et al. 2012) similar to the experimental observations. Whether the slow preparation region with increased activity is precursor and universal to the mainshock is still debated (Roeloffs 2006). In some cases, larger foreshocks were produced within these regions (Brodsky and Lay 2014). When, where and if these foreshocks occur are unknown and believed to be caused by heterogeneity in fault properties in space (e.g., Tesei et al. 2014; Luo and Ampuero 2018; Bedford et al. 2022). Models have been proposed to understand the interplay between precursory seismicity and the impending mainshock (e.g., Noda et al. 2013; Selvadurai et al. 2023); however, these rely on frictional parametrization and the spatial distribution of the heterogeneity. Both seismicity and slow slip represent dissipative processes in the crust. Models that can explain these phenomena, which also self-localize within a volume and produce accurate dynamics, are necessary to understand the nucleation processes. However, no models currently exist that fully explain the hydro-seismo-mechanical response of these systems. Developing computational tools that accurately predict regions that are suspect to localize strain is a first step, directly taken here.

### Various Patterns of Strain Localization

#### Detecting Strain Localization From the Bulk Deformation

Strain localization is detected when specific volumes of rock experience higher deformation with respect to rest of the sampled formation and, consequently, the standard deviation of the strain measurements retrieved increases. This phenomenon was observed in both the laboratory (Fig. 4) and numerical (Fig. 8) macroscopic deformations, where the standard deviations related to the axial, circumferential and volumetric strains increased with increasing differential stress. The computational model demonstrated its ability to simulate preparatory processes related to strain localization, correlating well with the DSS measurements obtained during the test. Differences between the laboratory and numerical results were remarked in the axial measurements, with the computational model showing only a slight increase of the standard deviation towards the sample failure. The circumferential and volumetric deformation behaviors of the numerical model correlated with the laboratory observations, with a significant increase of the range of strain measurements retrieved. Prior laboratory efforts similarly highlighted that triaxial experiments experienced maximum strain localization beyond 75% of the failure stress (McBeck et al. 2022b) and Ben-Zion and Zaliapin (2020) observed maximum localization of seismicity shortly prior to the major earthquakes in southern and Baja California. Our computational and laboratory observations agreed with these studies, as our measurements showed an increase of localization leading up to the failure. Within the elastic regime, the strain values determined in the simulations did not show any variation along the sample boundaries, deviating from our laboratory results. This indicates a perfectly homogeneous loading of the sample, achievable only with perfect parallelism of the two sample ends—a condition rarely met in laboratory experiments due to the technical limits of the specimen preparation process.

#### Accelerated Behavior

The AE and volumetric strain rates were shown to be linked (see Sect. 5.1) and, prior to the sample failure, we observed a simultaneous acceleration of both quantities (Fig. 10a). Similar accelerating dynamics were already observed in past laboratory efforts focused on the investigation of large stick–slip failure, where both the AE and strain rates showed to follow a power–law distribution preceding failure that might have been indicative of a run-away process (Dresen et al. 2020). In the field, coastal GPS sensors have been used to investigate the Mw 8.2 Iquique earthquake and showed preparatory deformation at large scale that subsequently lead to the mainshock (Bürgmann 2014; Socquet et al. 2017). Repeating earthquakes were also used to highlight an acceleration of preslip in the preparatory zones of the Mw 8.2 Iquique (Kato and Nakagawa 2014) and Mw 9.0 Tohoku-Oki (Kato et al. 2012) earthquakes. Figure 10b shows a comparison between the laboratory AE and volumetric strain rates and the mean dissipation determined in the regions of interest (ROI) (Fig. 9a) during the simulations as a function of time. During the majority of the triaxial test, both simulated and laboratory data showed a slightly increasing linear trend with time. The computational model captured an accelerating process only shortly prior to the nucleation of the macrofracture, which resembled the one detected by the AE and volumetric strain rates.

#### Dissipation Fronts

**Fig. 11:**
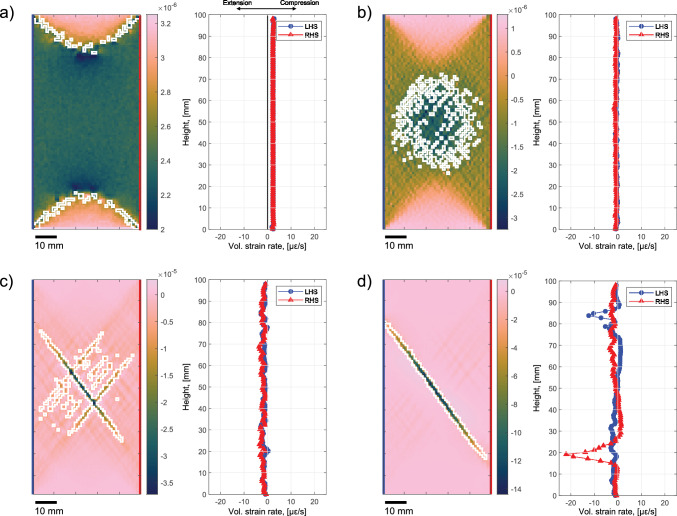
The same snapshots at **a** $t_{step}$ = 175, **b** 400, **c** 561 and **d** 571 shown in Fig. 9a were provided, where the ROIs of highly dissipative elements (white mask) were superimposed on the volumetric strain rate fields. For each snapshot, we also plotted the volumetric strain rate on the outer most elements on the left-hand side (LHS, blue) and right-hand side (RHS, red) of the sample

**Fig. 12:**
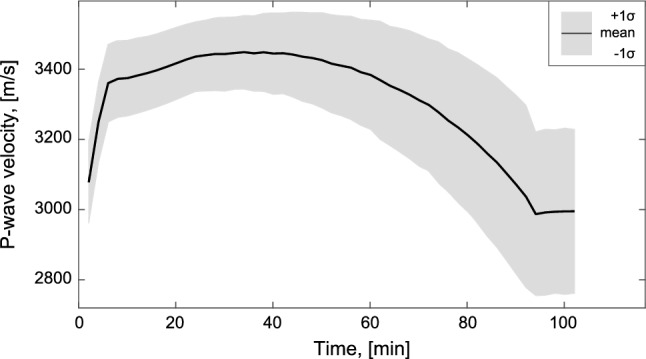
Time-varying homogeneous P-wave velocity model with respective uncertainties

The high dissipation fronts observed in the left panel of Fig. 9a showed to propagate from the sample ends towards the center during approximately the first half of the simulations (stage 1). The direction of the propagation and the location of these fronts correlated spatially with the same trend observed with the AE locations (Fig. 5c). The latter showed to migrate towards the sample center during the test before localizing on one side and develop into the macrofracture. Propagating fronts of AEs, which were interpreted as damage fronts, were already observed during triaxial experiments on sandstones (e.g., Lockner et al. 1992; Lockner 1993; Lei and Ma 2014). In Fig. 11 we displayed the volumetric strain rate field at the same snapshots used in Fig. 9 and we superimposed the ROI of the highly dissipative elements discussed in Sect. 4.2. Provided were also the measurements of the volumetric strain rate retrieved in the simulations along the outer most elements on the left-hand side (LHS, blue) and right-hand side (RHS, red) of the sample.

The dissipation fronts enclosed regions with higher compressional rates, compared to the rest of the sample, which grew as the fronts propagated towards the center. This compressional behavior combined with the propagation direction could indicate the occurrence of compaction bands at the sample ends (Menéndez et al. 1996; Fortin et al. 2005; Wong and Baud 2012; Brantut 2018). Even though the PZT sensors and the model detected deformation in these regions, the laboratory DSS data showed only a slight increase in the detected measurements (Fig. 10b). Similarly, the mean dissipation presented in both Figs. 9b and 10b slightly increased during the propagation of the fronts. Both the measurements retrieved with the optical fibers and numerical strains sampled on the RHS and LHS of the specimen during the simulations (right panel in Fig. 11a) did not show any form of strain localization. The computational model appeared to correctly capture the physical processes occurring at the two sample ends during this stage of the test and the modeled deformation behavior correlated with the laboratory observations.

#### Preparatory Process in the Central Region of the Sample

During stage 2, we observed a localization of high dissipative regions in the middle of the sample (second panel in Fig. 9a) quantified by the simultaneous drop of the number of contiguous regions with an increase of the area fraction (Figs. 9c, d). We notice that these ROI predominantly exhibited strong extensional behavior, indicative of dilation (Fig. 11b). While this region did not experience seismic deformation, the simulated preparatory process spatio-temporally correlated with the decrease of seismic velocity presented in Sect. 3.4. A possible explanation for such velocity anomalies, which is also sustained by the microscopical observations provided in Figs. 7b–e, could be the accumulation of damage—a process that could also occur aseismically (Cartwright-Taylor et al. 2022) and that has been shown to cause significant velocity drops (e.g., Scott et al. 1993; Paterson and Wong 2005; Pellet and Fabre 2007). Seismic velocity changes were also observed in the field by analyzing travel time perturbations in the coda of the correlation functions (Campillo and Paul 2003; Obermann and Hillers 2019). These changes were associated with damage and rupturing processes occurring in the years following the 2003 Mw 6.5 San Simeon and the 2004 Mw 6.0 Parkfield (Brenguier et al. 2008) earthquakes and during the months both prior to and after the 2009 Mw 7.1 Ridgecrest earthquake (Boschelli et al. 2021). Laboratory studies based on dynamic X-ray tomography have shown the ability to capture damage occurring during progressive failure of rock samples in triaxial settings and highlighted that, to thoroughly quantify deformation, it is not sufficient to only rely on seismic detection techniques (Cartwright-Taylor et al. 2022). As also captured by H-MEC and with the ultrasonic measurements, their results confirmed that aseismically preparatory processes could be detected in the sample prior to failure. Even though the volumetric strain rate profiles computed on the LHS and RHS of the sample (Fig. 11b) appeared to be slightly more noisy than in stage 1, these were still predominantly homogeneous without showing significant evidence for strain localization on the surface.

#### Preparation of a Weak Plane

In the last stage of the simulation we observed a system of conjugate bands that first formed (third panel of Fig. 9a) and then collapsed to a single band (fourth panel of Fig. 9a). The volumetric strain rate fields at the same snapshots (Figs. 11c, d) highlighted that these bands were dominated by dilation. We interpreted the growth of the single band before the sample failure as the preparation of a weak plane, which finally developed into the macrofracture. In the field, it has been shown that large earthquakes can rupture along weakened planes that were prepared by the accumulation of damage during the years preceding the main rupturing events (Ben-Zion and Lyakhovsky 2019; Ben-Zion and Zaliapin 2020; Ben-Zion and Ampuero 2009). At the two extremities of the simulated weak plane (i.e., within the process zone of the plane), we noted that dilation was localized under the shape of lobes. As the weak plane approached the surface, the RHS and LHS volumetric strain rate profiles showed localization in correspondence to the position of these lobes and were comparable to the laboratory DSS measurements (Fig. 6e). Due to these close similarities, we believe that the AE localization observed prior to the macrofracture nucleation could have been caused by the interaction between the sample surface and the propagation of these dilational lobes within the process zone of the weak plane. In the simulations we observed strain localization on the sample surface on both extremities of the weak plane. We could not observe the same level of symmetry in the DSS measurements, likely because of the presence of heterogeneities within the sample that favored the asymmetric development of the weak plane to only one side of the specimen.

Laboratory experiments have shown nucleation processes with accelerated localization of deformation on thin analog faults (Ohnaka and Shen 1999; Dieterich 1979; Selvadurai and Glaser 2015; Latour et al. 2013; McLaskey and Kilgore 2013). The empirical rate-and-state friction law has been effective in showing that a region that hosts preparatory slip will accelerate prior to the nucleation (Dieterich 1979; Ampuero and Rubin 2008). However, these models require thin, preassigned discontinuities. While effective in studying various aspects of the earthquake cycle, they are limited by the defined pre-existing geometries of the fault. Having models that are able to localize planes of weakness and produce the quasi-dynamic acceleration phase, as computed by H-MEC, may be useful to produce results that suffer less from user induced bias also at the larger reservoir or field scales. However, due to intrinsic damping routines of the computational solver (Gerya 2019), our numerical results are deemed reliable only up to the onset of fracture nucleation; dynamic propagation is not currently considered in this study.

### Implications of Coupled Process Modeling for Reservoir Scale Applications

Understanding the interplay between fluid and rock phases in geomechanical environments is crucial for scaling laboratory observations to reservoir applications. H-MEC integrates poro-elasto-visco-plastic relations to effectively simulate the complex interactions that occur under varied geophysical conditions, such as those induced by fluid injection in geothermal or petroleum extraction sites (Yarushina and Podladchikov 2015; Gerya 2019; Yarushina et al. 2020). These interactions often involve complex coupled processes that must be accurately modeled to prevent operational risks like induced seismicity (Moein et al. 2023).

The necessity of considering these coupled processes in numerical simulations is underscored by various studies that have used similar computational tools to model dynamic geological conditions. For instance, applications to crystalline rock formations at the reservoir scale have demonstrated the ability of computational models to predict heterogeneous fluid pressure fronts and associated seismic deformations (Petrini 2019; Bianchi 2020). However, limitations exist in models that lack multi-phase formulations, as seen in a previous study, which could not account for pore space reactivity due to the absence of a crucial fluid phase (Bianchi et al. 2022). For this reason, such models are limited to scenarios that do not involve significant rock–fluid interactions. Furthermore, the transition from brittle to ductile behavior, influenced by varying confining pressures, illustrates the need to include detailed viscosity-related parameters in simulations when applicable. This is particularly relevant in deeper geological settings where higher pressures may predominate (Bernabe and Brace 1990).

Various other modeling frameworks, such as the discrete element method (DEM) and the Particle Flow Code (PFC), have also been employed to study rock behavior under stress (McBeck et al. 2021a, 2022b; Wang and Cai 2019; Kulatilake et al. 2001; Koyama and Jing 2007). These models provide valuable insights into the fracture and fault development processes that are critical at the reservoir scale. For instance, DEM has been used to explore the effects of pre-existing fractures on fault network behavior, offering predictive insights into fault nucleation that are crucial for managing the risks associated with large-scale geomechanical projects. While the current discussion acknowledges the range of available numerical tools and their potential insights, it also highlights the specific advantages of H-MEC in handling the complexities observed in laboratory settings when scaled to larger geological frameworks. By focusing on the integration of hydro-mechanical and visco-elasto-plastic processes, H-MEC and similar models are well-positioned to enhance our understanding of nonlinear geomechanical behavior, which is crucial for effective resource extraction and geohazard management at the reservoir scale.

## Conclusions

This study investigated both aseismic and seismic preparatory processes linked to strain localization preceding rock failure. This was achieved by analysing the spatio-temporal deformation behavior of a sample of Berea sandstone during a triaxial experiment that employed a combination of novel DSS, AEs monitoring, ultrasonic surveys and physics-based numerical modeling. During the simulated experiment, we studied strain localization within the sample by tracking regions experiencing high dissipation of mechanical energy. Three stages were observed and used to explain the laboratory measurements: (i) highly dissipative fronts developed and propagated towards the middle of the sample similarly to the propagation trend observed with the AEs; (ii) in the second half of the experiment dissipative regions were produced in the middle of the sample, which spatio-temporally correlated with a discernible decrease of the P-wave velocities determined during the test; (iii) a system of conjugate bands formed and coalesced into a single band that grew from the center towards the sample surface. The latter was interpreted to be the preparation of a weak plane with dilatative lobes at the two process zones. Once these process zones interacted with the surface, it lead to strain localization and accelerated deformation observed both numerically and experimentally with the DSS array. This acceleration is believed to cause the observed increase in AE rate captured in a similar volume of the sample preceding the onset of dynamic failure. H-MEC appeared to capture a large variations of processes leading up to the nucleation of the shear fracture in a triaxial setting. Developing models that capture a range of behaviors at various scales, including the laboratory, is a necessary step to properly upscale research efforts to the reservoir and field scales. Application of the models should be treated with care as the current limitations of our numerical results include the inability to solve dynamic propagation, which will have implication on geohazards.

## Data Availability

The laboratory and numerical data used to investigate the triaxial test presented in this study are made open-source and can be retrieved from the following online repository: https://doi.org/10.3929/ethz-b-000611273 (Bianchi et al. 2023).

## Appendix A—Relevant Details Regarding the Acoustic Emission Analysis Workflow and P-Wave Velocity Model

A schematic of the workflow was provided in Bianchi et al. (2022), Fig. 4. In Sect. 2.4.1 we detailed how the AE data was reduced by picking detections with the STA/LTA algorithm and then by re-evaluating these picks with the AIC computational routine. We discarded classified events if the detections found were too sparsely separated in time. A theoretical maximal time delay between two detections due to the same AE always exists and equals the travel time between the two furthest PZT locations. If multiple passive detections from different channels were individuated in a time window with size equal or smaller to this maximal time delay, we evaluated this group of detections as belonging to a possible single AE. Consequently, we trimmed the waveforms in proximity of these detections and saved them separately for each possible AE individuated.

As the experiment finished, there was a single trace with AE measurements containing both passive and single events. To avoid interference from active shots on passive waveforms, we performed separate STA/LTA analyses on the segments of the waveforms that did not include ultrasonic surveys. This method ensured that the data analysis was carried out on clean segments of the waveforms, free from the influence of active pulses, thereby increasing the accuracy and reliability of the results. In this way we were able to discriminate between active and passive detections. To discard the passive detections individuated during the ultrasonic surveys, we manually selected the first active pulses of each PZT of the first survey and cross-correlated them with the rest of the picked active waveforms. We were able to retrieve approximately 95% of the total number of pulses. We aligned the active waveforms by individuating the cross-talk (i.e., the active pulse of the PZT shooting) with the AIC algorithm and we then stacked the shots together to improve the signal-to-noise ratio (SNR). We only accepted as reliable the waves that were detected at the receivers with an incident angle lower than 55$^\circ$. These results were then used to construct the time-varying homogeneous P-wave velocity models displayed in Fig. 12.

## Appendix B—Numerical Model Parameters

The initial parameters used in the simulations are listed in Table 1. The parameters initialized in the steel plates were either retrieved by available open-source tables listing stainless steel values or were assumed for convenience and to enhance the stability of the simulations. We assumed high values of shear viscosity and shear modulus and low values of porosity to avoid deformation occurring inside the plates during the simulations. Similarly, the cohesion and the internal friction coefficient were assumed to be very high so as to avoid yielding into the plates during the simulation. In the last millimeter of the steel plates towards the sample, we assumed an internal friction coefficient equal to 0 to remove nonphysical shear stresses from building up along the boundaries between these two materials. For simplicity, we initialized the confining medium with water parameters. The confining medium is only used to apply equal stress to the sample lateral sides. We expect differences on how the medium deforms (e.g., due to a different compressibility) between simulations with oil or water parameters but we assume no differences on how the stress is transferred to the sample. The physical processes occurring within the sample are thus independent from the parameter choice of the confining medium. To stabilize the simulations, the shear modulus was chosen to be equal to that of the sample, whereas the solid shear viscosity was assumed to be higher with respect to water but still a 15 order of magnitude smaller than the rock sample one. In this way no stress was built up by the confining medium when the differential stress was increased. For the same reasons mentioned above for the steel plates, we chose high values of the internal friction coefficient, cohesion and porosity. Permeability for all the three solid phases was assumed to be equal to 2$\times$10$^{-16}$ m$^2$, a value retrieved for Berea sandstone from Selvadurai and Suvorov (2022). The fluid phase used in the simulations was air, since the laboratory experiment was conducted on a dry sample. For numerical stability reasons, we assumed low fluid compressibility (i.e., water compressibility) of the pore medium and we compensated for it by imposing open sample boundaries for fluid outflow. This resulted in effective fluid escape and prevented fluid pressurization in the rock porous volume. Since we used the simulations to study the localization processes that occurred during a triaxial test on a intact rock sample, we decided to minimize the influence of rate-strengthening in the plasticity model by choosing a low value for the rate-strengthening exponent. For more details concerning the plasticity model choice, the reader will find exhaustive explanations in Dal Zilio et al. (2022).

**Table 1:** Numerical model initial parameters. $_\star$Determined experimentally, $_*$Open-source tables, $_\bullet$Value assumed, $_\diamond$Selvadurai and Suvorov (2022)

Parameter	Berea sample	Steel plates	Confining medium	Units
Solid density	$_\star$2120	$_*$7800	$_*$1000	kg/m$^3$
Solid shear viscosity	$_\bullet$10$^{25}$	$_\bullet$10$^{30}$	$_\bullet$10$^{10}$	Pa$\cdot$s
Shear modulus	$_\star$6.9$\times$10$^{9}$	$_\bullet$10$^{11}$	$_\bullet$6.9$\times$10$^{9}$	Pa
Solid compressibility	$_\star$9.5$\times$10$^{-11}$	$_*$6.1$\times$10$^{-12}$	$_*$4.5$\times$10$^{-10}$	Pa$^{-1}$
Cohesion	$_\star$18.0$\times$10$^{6}$	$_\bullet$10$^{9}$	$_\bullet$10$^{9}$	Pa
Int. friction coefficient	$_\star$0.58	$_\bullet$0.9	$_\bullet$0.9	–
Porosity	$_\diamond$0.19	$_\bullet$0.01	$_\bullet$0.01	–
Permeability	$_\diamond$2$\times$10$^{-16}$	$_\bullet$2$\times$10$^{-16}$	$_\bullet$2$\times$10$^{-16}$	m$^2$
Fluid density	$_*$1	$_*$1	$_*$1	kg/m$^3$
Fluid compressibility	$_*$4$\times$10$^{-10}$	$_*$4$\times$10$^{-10}$	$_*$4$\times$10$^{-10}$	Pa$^{-1}$
Fluid shear viscosity	$_*$10$^{-5}$	$_*$10$^{-5}$	$_*$10$^{-5}$	Pa$\cdot$s
Rate-strengthening exponent	$_\bullet$10$^{-3}$	$_\bullet$10$^{-3}$	$_\bullet$10$^{-3}$	–

The parameters chosen for the Berea sample in the simulations were largely determined by laboratory measurements or retrieved from Selvadurai and Suvorov (2022) (porosity and permeability). The density was calculated by its volume and weight and the elastic moduli from the Young’s modulus and Poisson’s ratio presented in Sect. 3.1. The shear viscosity was assumed to be higher than the typical Berea sandstone value, since, in the laboratory, the timescales are typically much lower with respect to the field scale. The internal friction angle was $\sim$30$^\circ$ and was determined from the angle of the main fracture (estimated from the AE locations) with respect to the axial axis of the sample and the cohesion was estimated by fitting the well-known Mohr-Coulomb law to three failure tests conducted on Berea sandstone samples at three different confining pressures: 10, 20 (presented here) and 40 MPa. The dilation angle was determined to be $\sim$15$^\circ$ from the volumetric and axial DSS measurements available. Randomized heterogeneities (±10%) from the values listed in Table 1 of the porosity, elastic moduli, cohesion and internal friction angle were initialized to better simulate heterogeneities in the sample driven by the interaction of different grain types.
